# From chaos to order: optimizing fecal microbiota transplantation for enhanced immune checkpoint inhibitors efficacy

**DOI:** 10.1080/19490976.2025.2452277

**Published:** 2025-01-18

**Authors:** Anqi Lin, Aimin Jiang, Lihaoyun Huang, Yu Li, Chunyanx Zhang, Lingxuan Zhu, Weiming Mou, Zaoqu Liu, Jian Zhang, Quan Cheng, Ting Wei, Peng Luo

**Affiliations:** a Department of Oncology, Zhujiang Hospital, Southern Medical University, 253 Industrial Avenue, Guangzhou, Guangdong, China; b Department of Urology, Changhai hospital, Naval Medical University (Second Military Medical University), Shanghai, China; c Department of Urology, Shanghai General Hospital, Shanghai Jiao Tong University School of Medicine, Shanghai, China; d Institute of Basic Medical Sciences, Chinese Academy of Medical Sciences and Peking Union Medical College, Beijing, 100730, China; e Department of Neurosurgery, Xiangya Hospital, Central South University, Changsha, Hunan, China; f National Clinical Research Center for Geriatric Disorders, Xiangya Hospital, Central South University, Hunan, China; g Cancer Centre and Institute of Translational Medicine, Faculty of Health Sciences, University of Macau, Macau SAR, 999078, China

**Keywords:** Fecal Microbiota Transplantation, immune checkpoint inhibitors, controversial effects, cancer immunotherapy, microbiome modulation

## Abstract

The integration of fecal microbiota transplantation (FMT) with immune checkpoint inhibitors (ICIs) presents a promising approach for enhancing cancer treatment efficacy and overcoming therapeutic resistance. This review critically examines the controversial effects of FMT on ICIs outcomes and elucidates the underlying mechanisms. We investigate how FMT modulates gut microbiota composition, microbial metabolite profiles, and the tumor microenvironment, thereby influencing ICIs effectiveness. Key factors influencing FMT efficacy, including donor selection criteria, recipient characteristics, and administration protocols, are comprehensively discussed. The review delineates strategies for optimizing FMT formulations and systematically monitoring post-transplant microbiome dynamics. Through a comprehensive synthesis of evidence from clinical trials and preclinical studies, we elucidate the potential benefits and challenges of combining FMT with ICIs across diverse cancer types. While some studies report improved outcomes, others indicate no benefit or potential adverse effects, emphasizing the complexity of host-microbiome interactions in cancer immunotherapy. We outline critical research directions, encompassing the need for large-scale, multi-center randomized controlled trials, in-depth microbial ecology studies, and the integration of multi-omics approaches with artificial intelligence. Regulatory and ethical challenges are critically addressed, underscoring the imperative for standardized protocols and rigorous long-term safety assessments. This comprehensive review seeks to guide future research endeavors and clinical applications of FMT-ICIs combination therapy, with the potential to improve cancer patient outcomes while ensuring both safety and efficacy. As this rapidly evolving field advances, maintaining a judicious balance between openness to innovation and cautious scrutiny is crucial for realizing the full potential of microbiome modulation in cancer immunotherapy.

## KEY POINTS

1. FMT’s impact on ICI efficacy varies across studies. Some show improved outcomes, others no benefit or adverse effects, highlighting complex host-microbiome interactions in cancer immunotherapy.
2. FMT affects ICI efficacy by modulating gut microbiome diversity, composition, and metabolites. It enhances T cell function and alters tumor microenvironment, but may promote immunosuppressive cells.
3. FMT outcomes depend on donor selection, recipient characteristics, and administration protocols. Varied responses necessitate personalized approaches considering individual microbiomes and tumor profiles.
4. Large-scale trials are needed to clarify FMT’s effects on ICI efficacy. Future research should optimize combinations, assess long-term safety, and use multi-omics for precise therapies.

## 1. Introduction

The gut microbiome, representing the largest symbiotic microbial community in the human body, exerts significant regulatory effects on the development and function of the host immune system through its metabolites and direct interactions. Research indicates that the gut microbiota modulates the immune system through various mechanisms, including regulation of innate and adaptive immune responses, orchestration of T cell differentiation and function, and facilitation of antigen presentation processes.^1^ With regard to innate immunity, gut microbes activate innate immune responses by stimulating pattern recognition receptors (PRRs), including Toll-like receptors (TLRs), NOD-like receptors (NLRs), and AIM2-like receptors.^2^ These receptors recognize microbial-associated molecular patterns (MAMPs), formerly known as pathogen-associated molecular patterns (PAMPs), triggering inflammatory responses and cytokine release. For instance, Han et al. demonstrated that Lactobacillus rhamnosus HDB1258, isolated from breastfed infant feces, enhances innate immune responses by activating macrophages and natural killer (NK) cells while promoting balanced expression of anti-inflammatory (interleukin-10, IL-10) and pro-inflammatory (tumor necrosis factor-alpha, TNF-α) factors.^3^ With respect to adaptive immunity, gut microbes exert substantial influence on the differentiation and function of T cells and B cells. Distinct microbial species can promote the differentiation of specific T cell subsets. For example, *segmented filamentous bacteria* facilitate the generation of T helper 17 (Th17) cells,^4^ while certain species of *Clostridium* promote the differentiation of CD4+ regulatory T (Treg) cells.^5^ The balance among these immune cell subsets is crucial for maintaining intestinal immune homeostasis. Furthermore, gut microbes modulate immune responses through their metabolites, particularly short-chain fatty acids (SCFAs).^6^

Immune checkpoint inhibitors (ICIs), including anti-programmed death receptor-1 (PD-1)/programmed death ligand-1 (PD-L1) and anti-cytotoxic T lymphocyte associated antigen-4 (CTLA-4) monoclonal antibodies, reinvigorate antitumor immune responses by blocking key pathways that tumor cells utilize to evade immune surveillance. These therapeutic agents have exhibited significant clinical benefits in a range of malignancies, including melanoma, non-small cell lung cancer, and colorectal cancer.^7–12^ PD-1, a type I transmembrane protein expressed on T cells, B cells, and NK cells, attenuates T cell activity upon binding to its ligand, PD-L1. CTLA-4, in contrast, diminishes co-stimulatory signals received by T cells through competitive binding with CD80/CD86. ICIs reinstate the immune-killing function of T cells by abrogating these inhibitory signals. However, clinical observations have indicated that the efficacy of ICI treatment is not universal.^13,14^ Emerging evidence suggests that this heterogeneity in treatment response may be intricately related to the composition of individual patients’ gut microbiomes. For instance, a seminal study demonstrated that melanoma patients exhibiting a response to ICI treatment possessed significantly higher gut microbial diversity compared to non-responders, and the abundance of specific bacterial taxa (such as *Clostridiales*) showed a positive correlation with treatment response.^7^ A subsequent study corroborated that patients responsive to anti-PD-1 therapy harbored a greater abundance of specific beneficial bacterial taxa (including *Bifidobacterium longum*, *Collinsella*, and *Enterococcus faecium*).^15^ These findings establish a theoretical foundation for augmenting the efficacy of ICIs through manipulation of the gut microbiome. Of particular significance, the administration of antibiotics may substantially impact the therapeutic efficacy of ICIs.^16–18^ A growing body of evidence indicates that the administration of antibiotics before or during ICI treatment is correlated with inferior clinical outcomes.^16–19^ This phenomenon may be attributed to antibiotics perturbing beneficial gut microbiota, thereby attenuating their modulatory effects on the immune system. Consequently, judicious use of antibiotics during ICI treatment, or implementing measures to reconstitute gut microbiota following necessary antibiotic administration, may represent a crucial strategy to enhance treatment efficacy.

Given the critical role of the gut microbiome in regulating antitumor immune responses, fecal microbiota transplantation (FMT), a therapeutic intervention that directly modulates the intestinal microbiome, has emerged as a promising adjunctive strategy to enhance the efficacy of ICIs.^20^ FMT involves the transfer of whole fecal material from a donor to a recipient to reconstruct the recipient’s gut microbial ecosystem, with the objective of preventing, treating, or alleviating specific clinical conditions.^21,22^ In addition to FMT, various therapeutic approaches for modulating the gut microbiome have emerged as potential strategies to enhance ICI efficacy. These include prebiotics (indigestible dietary compounds that promote beneficial gut microbiota), postbiotics (bioactive compounds produced through microbial metabolism that directly confer beneficial effects on the host), synbiotics (combinations of probiotics and prebiotics that act synergistically), and targeted interventions with specific bacterial strains.^23,24^ While each approach offers unique advantages, FMT represents the most comprehensive method for microbiome modulation, as it facilitates the transfer of complete microbial communities rather than individual components or specific interventions. FMT initially demonstrated efficacy in treating recurrent Clostridioides difficile infection (rCDI) and subsequently exhibited therapeutic potential in various other pathological conditions.^25^ In recent years, numerous clinical investigations have been initiated to evaluate the application of FMT in augmenting the efficacy of ICIs. Several studies have yielded promising results. For instance, a clinical trial conducted FMT in combination with ICI treatment in 10 patients with metastatic melanoma refractory to anti-PD-1 therapy, resulting in clinical responses or extended progression-free survival in a subset of 2 patients attained partial remission, 1 patient achieved complete remission, and 3 patients exhibited progression-free survival (PFS) exceeding 6 months.^26^ In a separate investigation, 40% of ICI-refractory melanoma patients demonstrated renewed responsiveness to ICIs following FMT. These findings indicate that FMT may augment the therapeutic effects of ICIs by modulating the gut microbiome. However, not all investigations corroborate the hypothesis that FMT can significantly enhance the clinical efficacy of ICIs. A clinical trial evaluating the SER-401 (comprising proprietary formulated bacterial Firmicutes spores isolated and purified from human donor stool) in melanoma patients, the combination treatment group failed to demonstrate significant clinical benefits when compared to nivolumab monotherapy.^27^ These discrepant outcomes underscore the conflicting effects of FMT in ICI treatment and emphasize the necessity for comprehensive investigation into the potential factors underlying these disparities. To date, there exists no comprehensive systematic review synthesizing the potential mechanisms underlying the inconsistent clinical benefits of FMT in ICI treatment.

In light of the potential and existing controversies surrounding the clinical benefits of FMT in combination with ICIs, this review will systematically explore the following aspects: First, we will conduct a comprehensive analysis of the controversial effects of FMT in tumor ICI therapy. By critically evaluating existing clinical research results, assessing inter-study variations, and synthesizing the impact of FMT on ICI efficacy, we aim to provide a comprehensive overview. Second, we will elucidate the potential reasons for the controversies surrounding FMT in ICI therapy, including the complexity of gut microbiota (encompassing factors such as composition and diversity), gut microbial metabolites, the impact of gut microbiota on the tumor microenvironment (TME) and signaling pathways, the heterogeneity of FMT techniques, and the influence of recipient or donor characteristics. Third, we will delineate the process of screening and optimizing FMT preparation. Fourth, we will address unresolved issues in current FMT applications, explore future research directions, and propose potential solutions. As our understanding of the role of the gut microbiome in tumor immunity continues to evolve, FMT, as a potential adjunctive therapy, demonstrates significant potential in enhancing the efficacy of ICIs. However, the clinical application of FMT still faces numerous challenges and controversies. By systematically synthesizing existing research findings, comprehensively analyzing the reasons for controversies, and proposing future research directions, this review aims to facilitate the further development and optimization of FMT in cancer immunotherapy, potentially contributing to improved treatment outcomes and quality of life for cancer patients.

## 2. Controversial effects of FMT on ICI therapy

FMT, an emerging therapeutic modality, has demonstrated considerable promise in augmenting the efficacy of ICIs; however, the clinical effectiveness of this approach remains a subject of debate. In recent years, a multitude of clinical studies have focused on elucidating the impact of FMT on ICIs treatment outcomes (Table 1); however, the research findings exhibit considerable heterogeneity.

**Table 1.** Summary of clinical trials investigating the integration of fecal microbiota transplantations (FMT) with Immune Checkpoint Inhibitors (ICIs) therapy.

Tumor Types	Clinical Trial Number	Trial Phase	Donor	Number of Recipients	Immunotherapy	Primary Outcome Measures	Approaches of FMT
MSS-mCRC	ChiCTR2100046768	NA		20	Anti-PD-1 therapy+Anti VEGFR	PFS	FMT (via stool capsules)
Melaoma	NCT03341143	phase I	Responders to ICIs	15	Pembrolizumab	ORR	FMT (via colonoscopy)
Melaoma	NCT03353402	phase I	Responders to ICIs	10	Anti-PD-1 therapy	AEs, Proper implant engraftment	FMT (via colonoscopy and stool capsules)
Melaoma	NCT03772899	phase I	Healthy People	20	Nivolumab/Pembrolizumab	Measure of Safety	FMT (via stool capsules)
Melaoma	NCT03817125	phase Ib		14	Nivolumab	AEs	SER-401 (oral formulation enriched with Thickettsia spores)/Placebo; FMT
Solid Tumors	NCT03819296	phase I/II	Healthy People	54	Infliximab/Vedolizumab	Difference in stool microbiome pattern, AEs	FMT(via endoscopy)
RCC	NCT04038619	phase I	Healthy People	7	ICIs+Loperamide	AEs, Clinical Response, Remission of immune-related diarrhea/colitis	FMT (via colonoscopy)
Mesothelioma	NCT04056026	phase I	Healthy People	1	Keytruda	PFS	FMT (via colonoscopy)
Prostate Cancer	NCT04116775	phase II		32	Enzalutamide + Pembrolizumab		FMT(via endoscopy)
Gastrointestinal Cancer	NCT04130763	phase I	Healthy People	10	Anti-PD-1 therapy	ORR, Rate of abnormal vital signs and laboratory test results, AEs	FMT (via stool capsules)
mRCC	NCT04163289	phase I	Healthy People	10	Ipilimumab/Nivolumab	AEs	FMT (via stool capsules)
Solid Tumors	NCT04264975	NA	PR/CR Responders to ICIs	13	Anti-PD-1 therapy	ORR	FMT (via endoscopy)
Melaoma, NSCLC	NCT04521075	phase I/II	CR Responders to ICIs	42	Nivolumab	AEs/ORR	FMT (via stool capsules)
Melaoma	NCT04577729	phase II (Terminated)		60	Pembrolizumab/Nivolumab	Measure of Safety	Allogeneic FMT/Autologous FMT
CRC	NCT04729322	phase I	Responders to ICIs		Pembrolizumab/Nivolumab	ORR	FMT (via stool capsules)
RCC	NCT04758507	phase I/II	Responders to ICIs	50	ICIs	Clinical Benefit (SD/PR/CR)	FMT (via stool capsules)
Solid Tumors	NCT04883762	phase I	Healthy People	4	ICIs	AEs	FMT (via endoscopy)
Advanced Lung Cancer	NCT04924374	NA	10 healthy People with High-fiber Diets (>30 g/day)	20	Anti-PD1 (Pembrolizumab, Nivolizumab, Atezolizumab)	Measure of safety	FMT (via stool capsules)
Melaoma, NSCLC	NCT04951583	phase II	Responders to ICIs	20	Pembrolizumab/Nivolumab/ipilimumab	Measure of Safety	FMT (via stool capsules)
NSCLC	NCT05008861	phase I		20	Anti-PD-1/PD-L1 therapy+Platinum based chemotherapy	AEs	FMT (via stool capsules)
Melanoma Stage III Melanoma Stage IV	NCT05251389	phase Ib/IIa	Responders to ICIs/Non-responding to ICIs	24	ICIs	Clinical Benefit(SD/PR/CR)	FMT (via endoscopy)
Pan-cancer (stage IV)	NCT05273255	NA	CR Responders to ICIs with minimum duration ≥12 months	30	ICIs	Mean change from baseline of bacterial species	FMT (via colonoscopy)
CRC	NCT05279677	phase II		30	Sintilimab + Fruquintinib	Change in the intestinal microbiome	FMT (via stool capsules)
Melanoma, Head and Neck Squamous Cell Carcinoma, Cutaneous Squamous Cell Carcinoma, (MSI-High), Clear Cell RCC, NSCLC Cancer	NCT05286294	phase IIa	Responders to ICIs	20	ICIs	AEs, Clinical Benefit(SD/PR/CR)	FMT
Metastatic Lung Cancer	NCT05502913	phase II	PR/CR Responders to ICIs	80	Chemo-Anti-PD-L1 therapy	PFS	FMT(via stool capsules)
Solid Tumors	NCT05533983	NA	Responders to ICIs remaining in CR/PR/SD for at least 6 months according to RECIST v1.12	50	Nivolumab	ORR	FMT
Advanced HCC	NCT05690048	phase II（Not yet recruiting）		48	Atezolizumab + Bevacizumab	Differential tumoral CD8 T-cell infiltration, AEs	FMT (via stool capsules)
Advanced HCC	NCT05750030	phase II		12	Atezolizumab + Bevacizumab	safety of FMT	FMT
Advanced Gastric Cancer	NCT06346093	NA		66	Anti-PD-1 therapy/Chemotherapy	ORR, Clinical Benefit(SD/PR/CR)	FMT (via stool capsules)

FMT: Fecal Microbiota Transplantation, PR: Partial Response, CR: Complete Response, SD: Stable Disease, PD-1: Programmed Cell Death Protein 1, PD-L1: Programmed Death Ligand 1, NSCLC: Non-Small Cell Lung Cancer, RCC: Renal Cell Carcinoma, CRC: Colorectal Cancer, AEs: Adverse Events, ORR: Objective Response Rate, PFS: Progression-Free Survival, OS: Overall Survival, MSS: Microsatellite Stability, MSI: Microsatellite Instability; SER-401: comprising proprietary formulated bacterial Firmicutes spores isolated and purified from human donor stool; ICIs: Immune Checkpoint Inhibitors; HCC: Hepatocellular Carcinoma; VEGFR: Vascular Endothelial Growth Factor Receptor; Chemo: Chemotherapy.

### 2.1. Potential benefits of FMT on ICI treatment efficacy: current evidence

#### 2.1.1. Melanoma

Emerging clinical evidence indicates that FMT may substantially augment the therapeutic efficacy of ICIs in patients with melanoma. In a prospective clinical trial (NCT03341143) involving patients with advanced melanoma, investigators administered FMT intervention to 15 subjects concurrently receiving pembrolizumab treatment.^28^ The results demonstrated that 40% of patients experienced clinical benefit and manifested rapid and sustained alterations in their gut microbiota. The gut microbiota of responders exhibited specific bacterial communities associated with anti-PD-1 response, concomitant with enhanced activation of CD8+ T cells and diminished myeloid cells, indicating that FMT may potentiate ICI efficacy by modifying the immune microenvironment. In a separate phase I clinical trial (NCT03353402), investigators administered treatment to 10 patients with anti-PD-1-refractory metastatic melanoma who had previously attained complete response (CR) following nivolumab monotherapy, and assessed the safety and feasibility of nivolumab re-induction.^29^ Three subjects attained six-month progression-free survival, with two exhibiting partial response (PR) and one achieving CR. Subsequently, the investigators performed comprehensive microbiome analysis and tissue biopsies. Overall, substantial alterations in gut microbiome composition were observed across all FMT recipients; the responder group demonstrated microbial characteristics conducive to immunotherapy, characterized by elevated relative abundances of *Enterococcaceae*, *Enterococcus*, and *Streptococcus australis*, and diminished relative abundance of *Veillonella atypica*. Advantageous modulations in immune cell infiltration and gene expression profiles were detected in the intestinal lamina propria the TME. A phase I clinical trial (NCT03772899) evaluating the combination of FMT from healthy donors (HD) with anti-PD-1 therapy in treatment-naive patients with advanced melanoma demonstrated objective responses in 13 patients (65%), comprising 4 (20%) CR and 9 (45%) PR. After a median follow-up of 20.7 months, the median progression-free survival remained unreached, with 16 patients maintaining survival^30^ The study also demonstrated durability of treatment responses, with a subset of patients exhibiting sustained clinical responses following the administration of combined FMT and anti-PD-1 therapy.

#### 2.1.2. Colorectal cancer

In a clinical trial (ChiCTR2100046768) focusing on patients with microsatellite stable metastatic colorectal cancer (MSS-mCRC), investigators implemented a triple therapy regimen combining FMT with PD-1 inhibitors and vascular endothelial growth factor receptor (VEGFR) inhibitors.^31^ Preliminary results suggested that this innovative triple therapy regimen effectively mitigated tumor progression, significantly decreased serum tumor marker levels, augmented patient immune function, and facilitated a balanced state of the gut microbiota. Gut microbiome analysis demonstrated that treatment responders harbored higher abundances of *Proteobacteria* and *Lachnospiraceae*, while exhibiting relatively lower abundances of *Actinobacteria* and *Bifidobacterium*. Although FMT did not significantly modify the overall structure of the peripheral blood T cell receptor (TCR) repertoire, clonally expanded TCR sequences displaying characteristics of antigen-driven immune responses were detected in treatment responders. In the NCT04951583 clinical trial, the objective response rate (ORR) post-FMT treatment attained 70%, comprising 2 cases of CR and 12 cases of PR. Further analysis elucidated significant differences in gut microbiome characteristics between responders and non-responders. Presently, multiple clinical trials investigating ICIs in combination with FMT treatment strategies are ongoing, including NCT04038619,^32^ NCT04163289,^33^ NCT04951583,^34^ and NCT05008861,^35^ which will further elucidate the potential of FMT in augmenting ICI efficacy.

#### 2.1.3. Other solid tumors

A subsequent study focusing on patients with specific solid tumors including metastatic gastric cancer (GC), esophageal squamous cell carcinoma (ESCC), and hepatocellular carcinoma (HCC) (NCT04264975) provided additional evidence supporting the positive role of FMT.^36^ Among 13 patients receiving anti-PD-1 therapy, FMT induced sustained microbiome changes and clinical benefits in 6 patients (46.2%), with one achieving partial response and five maintaining stable disease. The investigators observed significant increases in the levels of cytotoxic T lymphocytes (CTLs) and pro-inflammatory cytokines in both patients’ peripheral blood and the TME. Notably, *Prevotella merdae* isolated from the fecal samples of FMT responders was shown to enhance T cell activity by promoting CTLs infiltration and to significantly inhibit tumor growth in mouse xenograft models.

### 2.2. FMT failing to enhance clinical benefits of ICIs: existing evidence

However, not all studies corroborate the hypothesis that FMT can significantly improve the clinical benefits of ICIs. In a randomized controlled clinical trial (NCT03817125),^27^ researchers evaluated SER-401, a proprietary formulation of bacterial Firmicutes spores isolated and purified from the stool of human donors exhibiting favorable microbiome signatures associated with anti-PD-1 response. Although the study was initially designed as a four-arm trial encompassing both SER-401 and FMT interventions, it was subsequently modified to a two-arm placebo-controlled design owing to recruitment challenges during the COVID-19 pandemic. The implementation of vancomycin preconditioning in the study protocol introduced additional complexity to the interpretation of the results. Although SER-401 partially restored gut microbiome diversity after vancomycin pretreatment, the objective response rate in the SER-401 group (25.0%) was lower than in the placebo group (66.7%). The disease control rate (DCR) in the SER-401 group was 37.5%, which was also lower than the 83.3% observed in the placebo group. Moreover, the PFS and overall survival (OS) in the SER-401 group exhibited a numerical downward trend. Although these differences did not reach statistical significance, the results highlight the complexity of microbiome modulation approaches in cancer immunotherapy. Subsequent immunological analysis revealed the presence of Tregs and effector T cells with high expression of T cell immune receptor with Ig and ITIM domains (TIGIT) and PD-1 in patients from the SER-401 group, indicating that the TME may have shifted toward a state less conducive to anti-tumor immune responses. Sequencing analysis of immune-related gene expression profiles further revealed that patients in the SER-401 group exhibited increased activation of signaling pathways associated with immune checkpoint blockade (ICB) resistance. Notably, the NCT03817125 trial has been completed, and the findings have provided valuable insights into targeted microbiome interventions for cancer immunotherapy. This trial represents the most recent and only clinical evaluation of SER-401, highlighting both the promise and challenges in translating microbiome-based approaches to clinical practice.

The etiology underlying the controversial effects of FMT on ICI treatment efficacy is likely multifactorial. At present, the academic literature lacks comprehensive reviews and rigorous analyses elucidating the reasons for the controversial effects of FMT on ICI treatment efficacy. While the impact of FMT on ICI efficacy remains a subject of debate, it is imperative to recognize that this nascent research domain is still in its formative stages. Future investigations should prioritize the implementation of large-scale, multi-center, randomized controlled clinical trials to comprehensively assess the impact of FMT on ICI treatment efficacy and elucidate potential predictive biomarkers. As our comprehension of the mechanisms underlying FMT-mediated modulation of the tumor immune microenvironment advances, and as we refine FMT preparation and administration protocols, it is reasonable to anticipate that future applications of FMT will be more precise in augmenting the anti-tumor effects of ICIs, potentially yielding substantial clinical benefits for patients with cancer.

## 3. Potential mechanisms underlying varied clinical benefits of ICIs following FMT

**Figure 1.**
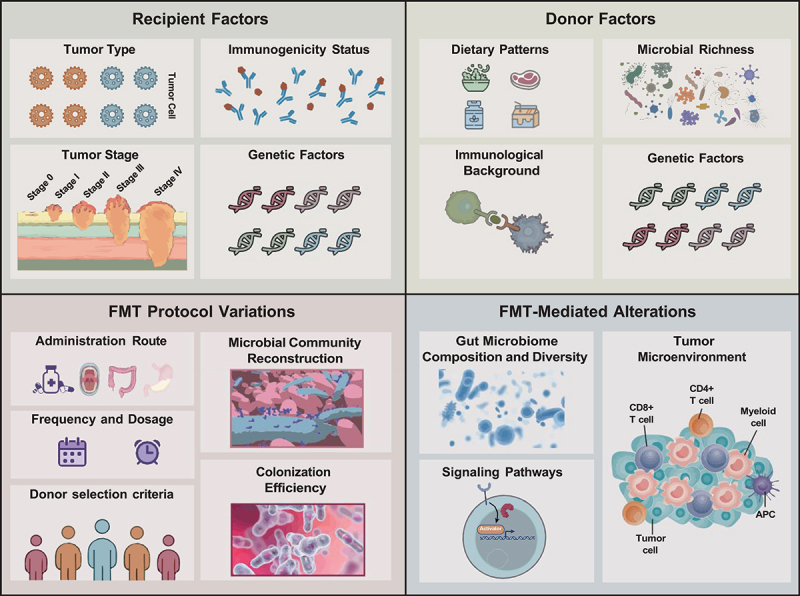
Key factors influencing the therapeutic efficacy of fecal microbiota transplantation (FMT) in combination with Immune Checkpoint Inhibitor (ICI) therapy.

**Figure 2.**
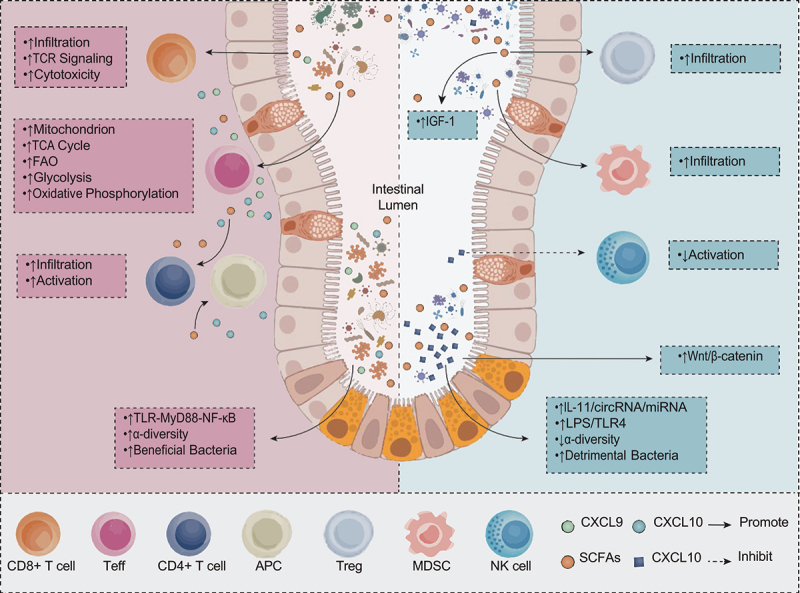
Mechanisms by which fecal microbiota transplantation (FMT) influences the therapeutic efficacy of immune checkpoint inhibitors (ICIs).

The factors contributing to the discrepancies in the effects of FMT on ICI treatment efficacy are multifaceted. FMT potentially modulates the clinical benefits of ICIs through various mechanisms (Figures 1–2). Beyond directly altering gut microbiota composition and diversity, FMT can potentially affect the clinical benefits of ICIs through microbial metabolites. Furthermore, FMT has the potential to influence the recipient’s immune cell function, the TME, and intracellular signaling pathways. Moreover, FMT protocols utilized in different studies exhibit variability in administration routes, frequency, dosage, and donor selection criteria. These factors can influence the colonization efficiency of FMT, the extent of microbiome reconstitution, and consequently, its modulatory effect on ICI efficacy. Individual differences in recipients, including tumor type, stage, and immunogenicity status, can impact the modulatory effect of FMT on ICI efficacy. Donor-specific factors, encompassing dietary patterns, microbiome richness, immunological background, and genetic factors, can also modulate the effect of FMT on ICI efficacy.

This figure delineates the major factors influencing the therapeutic efficacy of FMT when used in conjunction with ICI therapy. These factors are stratified into four main categories: donor-related factors, recipient-related factors, FMT protocol parameters, and FMT-mediated alterations. Donor-related factors encompass dietary patterns, microbial richness, immunological background, and genetic factors, which collectively influence the quality and potential efficacy of the donor fecal material. Recipient-related factors comprise tumor type and stage, immunogenicity status, individual variations, and genetic factors, which modulate the patient’s response to FMT and ICI therapy. The FMT protocol encompasses administration route, frequency and dosage, colonization efficiency, microbial community reconstruction, and donor selection criteria, which directly impact the implementation and efficacy of FMT. FMT-mediated alterations primarily comprise changes in gut microbial composition and diversity, microbial metabolites, and the tumor microenvironment, which represent the direct mechanisms by which FMT modulates the efficacy of ICI therapy. A comprehensive consideration of these factors facilitates a better understanding of the impact of FMT on the therapeutic efficacy of ICI therapy, thereby guiding future research and clinical applications.

FMT: Fecal Microbiota Transplantation; ICI: Immune Checkpoint Inhibitor;

This figure illustrates the multifaceted mechanisms by which FMT modulates the therapeutic efficacy of ICIs. The figure delineates key cellular and molecular events occurring in the intestinal lumen, intestinal epithelium, and tumor microenvironment. FMT may augment the clinical benefits of ICIs through various mechanisms, including but not limited to the following. In the intestinal lumen, FMT can elevate α-diversity and promote beneficial bacterial populations. This microbial modulation affects the functionality and abundance of various immune cells. Consequently, CD8+ T cell infiltration is augmented, TCR signaling is potentiated, and cytotoxicity is heightened. Additionally, the infiltration and activation of CD4+ T cells and APCs are enhanced. The metabolism of Teffs undergoes substantial alterations, including elevated mitochondrial numbers, augmented TCA cycle activity, enhanced FAO, intensified glycolysis, and amplified oxidative phosphorylation. These metabolic adaptations provide T cells with increased energy reserves, thereby enhancing their anti-tumor function. At the molecular level, FMT can induce the activation of the TLR-MyD88-NF-κB signaling pathway. FMT can also upregulate the expression of chemokines CXCL9 and CXCL10, promoting T cell migration to tumor sites. While FMT can potentiate the efficacy of ICIs in some cases, it may not confer improved ICI efficacy in certain patients. For instance, following FMT, the recipient’s pathogenic bacteria may proliferate while α-diversity diminishes. Moreover, SCFAs, microbial metabolites, further enhance the infiltration of Tregs and MDSCs, while promoting the upregulation of IGF-1 expression. FMT may also potentiate the regulation of the Wnt/β-catenin signaling pathway through microbial metabolites, notably DCA, thereby modulating tumor cell growth and metastasis. Furthermore, DCA suppresses the activation of NK cells. FMT may also augment the activity of the IL-11/circRNA/miRNA axis and the LPS/TLR4 axis, thereby facilitating tumor progression. These intricate interactions cumulatively determine the impact of FMT on ICIs treatment efficacy, elucidating why certain patients experience enhanced ICI efficacy after FMT, while others may not exhibit significant changes.

FMT: Fecal Microbiota Transplantation; ICI: Immune Checkpoint Inhibitor; TCR: T cell receptor; APCs: antigen-presenting cells; Teffs: effector T cells; TCA: tricarboxylic acid; FAO: fatty acid oxidation; SCFAs: short-chain fatty acids; Tregs: regulatory T cells; MDSCs: myeloid-derived suppressor cells; IGF-1: insulin-like growth factor-1; DCA: deoxycholic acid; NK: natural killer

### 3.1. FMT-Mediated alterations in Host physiology

#### 3.1.1. Modulation of gut microbiota diversity

Alterations in gut microbial diversity represent one of the key factors underlying variations in ICI treatment efficacy following FMT. Accumulating evidence indicates that the diversity of the gut microbiome is closely associated with the therapeutic response to ICIs. Gut microbial diversity has been demonstrated to potentially enhance the efficacy and prognosis of ICI treatment. Research indicates that high α-diversity of the gut microbiota correlates with favorable responses to ICI treatment, potentially attributed to the ability of diverse microbiota to promote CD8+ T cell infiltration into tumors.^37,38^ A longitudinal study in HCC patients demonstrated temporal increases in gut microbiome diversity during immunotherapy treatment,^39^ though the direct correlation with treatment response requires further investigation. Conversely, patients with low microbial diversity or dysbiosis frequently exhibit poor responses to ICI treatment. However, patients undergoing ICI treatment are susceptible to microbiome dysbiosis, potentially resulting in reduced diversity and a shift toward a Gram-negative enterotype.^40^ FMT has the capacity to reverse microbiome dysbiosis by introducing healthy microbial communities and modifying microbial composition.^41–44^ This intervention has the potential to not only augment the effectiveness of immunotherapy but also mitigate adverse reactions to immunotherapy.^45^

#### 3.1.2. Composition of gut microbiota

Variations in gut microbial composition also significantly influence the efficacy and prognosis of ICI treatment following FMT. Research has demonstrated that FMT can significantly modify the composition of the gut microbiome. Post-FMT, the abundance of dominant intestinal bacteria increases significantly, while enteric pathogens associated with intestinal oxidative stress exhibit a marked decrease.^43^ More specifically, the enrichment of certain bacterial taxa demonstrates a strong correlation with positive responses to ICI treatment. For instance, increased proportions of *Bifidobacterium longum*, *Collinsella aerofaciens*, and *Enterococcus faecium* correlate with positive responses to immunotherapy.^46^ Similarly, increased abundance of *Faecalibacterium*, *Ruminococcus*, and *Akkermansia muciniphila* is linked to enhanced responses to anti-PD-1 therapy.^39,47–51^ These beneficial bacteria may mediate their effects through various mechanisms, including the promotion of T cell activation, enhancement of anti-tumor immune responses, and maintenance of gut health.^52,53^ The taxa significantly enriched in ICI responders both pre- and post-FMT predominantly belong to *Firmicutes* and *Actinobacteria*, while the depleted bacteria primarily belong to *Bacteroidetes* .^54–57^ These alterations in bacterial strains may contribute to improved ICI efficacy.

Conversely, the presence of certain bacteria may negatively impact the efficacy of ICIs. For instance, the enrichment of *Proteobacteria* (including *Escherichia coli*, *Shigella*, and *Klebsiella*), *Clostridium*, and certain members of *Actinobacteria* has been correlated with adverse reactions to ICI treatment.^58–60^ Interestingly, some bacterial species show variable associations with treatment outcomes. For example, *Akkermansia muciniphila* has been correlated with favorable responses to ICI treatment in some studies,^57,61^ while in others it has been associated with resistance.^62,63^ *Bacteroides* has also exhibited similar bidirectional effects. This complexity may result in individual variations in ICI treatment efficacy following FMT. It is important to note that different ICI regimens may exert varying effects on the commensal microbiota.^61,64^ For instance, responders to nivolumab-ipilimumab demonstrated enrichment of *Faecalibacterium, Bacteroides thetaiotaomicron, and Holdemanella filiformis*, while patients responding to pembrolizumab showed enrichment of *Dorea formicigenerans* .^61^ This observation may elucidate the differences in FMT outcomes for various ICI treatment regimens.

#### 3.1.3. Gut microbial metabolites

Gut microbial metabolites are instrumental in modulating the host immune system and the TME, potentially serving as a critical determinant in elucidating the disparities in ICI treatment efficacy following FMT. FMT has the potential to modulate the therapeutic effects of ICIs through alterations in the metabolite profile generated by the gut microbial community. Current evidence indicates that certain microbial metabolites can potentiate the antitumor effects of ICIs, while others may attenuate their action. This dichotomy offers a plausible mechanistic basis for the heterogeneity in patient responses to ICI treatment following FMT.

SCFAs are pivotal metabolites synthesized by gut microbiota via the fermentation of dietary fiber, predominantly comprising acetate, propionate, and butyrate. In preclinical mouse models, research has demonstrated that SCFA levels are significantly elevated in FMT-treated mice, with notable increases in the concentrations of butyrate, acetate, and caproic acid.^56,65,66^ SCFAs augment the therapeutic effects of ICIs via multifaceted mechanisms, encompassing various levels of immune regulation. Empirical evidence suggests that butyrate can substantially potentiate the cytotoxic activity of CD8+ T cells.^65–67^ More precisely, in the mouse models, butyrate can augment the antitumor cytotoxicity of CD8+ T cells by suppressing the DNA-binding protein 2 (ID2)-dependent interleukin-12 (IL-12) signaling pathway, consequently enhancing the therapeutic effect of anti-PD-1 therapy.^68^ Additionally, in the mouse models, butyrate has been shown to elevate the levels of histone 3 lysine 27 acetylation (H3K27ac) in the promoter regions of programmed cell death gene 1 (Pdcd1) and CD28 molecules in CD8+ T cells, upregulating the expression of PD-1 and CD28, and modulating TCR signaling pathways. These actions synergistically promote the expression of antitumor cytokines in cytotoxic CD8+ T cells, thus potentiating the effect of anti-PD-1 therapy.^65,67,69^ Research has demonstrated that SCFAs can augment the mitochondrial content in antitumor effector T cells and upregulate key metabolic pathways, including glycolysis, oxidative phosphorylation, tricarboxylic acid (TCA) cycle, and fatty acid oxidation (FAO). These metabolic alterations not only ensure adequate energy provision for immune cells but also significantly enhance the therapeutic efficacy of ICIs.^66,70^ In the mouse models, SCFAs not only serve as an energy source for immune cells but also facilitate the differentiation of CD8+ T cells into memory T cells and promote their long-term survival.^20,71^ This effect is crucial in sustaining a prolonged antitumor immune response.

The impact of FMT on ICI efficacy exhibits considerable heterogeneity, with some patients showing no improvement or potentially diminished responses. This has been demonstrated in the randomized controlled clinical trial NCT03817125, where patients receiving SER-401 (a proprietary formulation of bacterial Firmicutes spores isolated and purified from the stool of human donors) showed lower objective response rates (25.0% vs 66.7%) and disease control rates (37.5% vs 83.3%) compared to the placebo group. Furthermore, immunological analysis revealed increased presence of Tregs and effector T cells with high expression of TIGIT and PD-1 in the SER-401 group, suggesting a shift toward an immunosuppressive tumor microenvironment. These patients also exhibited increased activation of signaling pathways associated with immune checkpoint blockade resistance. This heterogeneity in treatment response can be attributed to the following factors. Under specific conditions, SCFAs can elicit immunosuppressive effects, primarily characterized by the following mechanisms: SCFAs, particularly butyrate, have the capacity to suppress immune responses through activating G Protein-Coupled Receptor 43 (GPR43) and promoting the differentiation of Forkhead Box P3-positive (Foxp3+) CD4+ Tregs.^65,72^ Excessive proliferation of Tregs can potentially suppress anti-tumor immune responses, thereby diminishing the therapeutic efficacy of ICIs. Elevated concentrations of butyrate and propionate can compromise B lymphocyte DNA recombination capacity via Histone Deacetylase (HDAC) inhibition, resulting in impaired local intestinal and systemic T cell-dependent and T cell-independent antibody responses.^65^ Under specific conditions, SCFAs can potentially facilitate tumor progression through the activation of certain signaling pathways. For instance, in prostate cancer models, SCFAs have been shown to promote tumor growth and metastasis through the activation of the Insulin-like Growth Factor 1 (IGF-1) signaling pathway.^73^ Following FMT, some patients can develop bile acid metabolism disorders, potentially precipitating a series of adverse effects: Certain secondary bile acids, such as Deoxycholic Acid (DCA), can enhance tumor cell proliferation and survival through the activation of the Wnt/β-catenin signaling pathway.^74,75^ Specific bile acids have the potential to suppress the function of anti-tumor immune effector cells, including Natural Killer T cells (NKT cells), thereby compromising the body’s anti-tumor immune surveillance.^76^ Consequently, the variability in ICI treatment efficacy following FMT likely reflects the balance between these beneficial and detrimental factors. For some patients, FMT can result in an increase in beneficial metabolites and a decrease in harmful metabolites, consequently augmenting the efficacy of ICIs. Conversely, for other patients, FMT can induce an increase in harmful metabolites or a decrease in beneficial metabolites, thereby attenuating the efficacy of ICIs.

#### 3.1.4. Alterations in the tumor microenvironment

FMT has the potential to augment the antitumor effects of ICIs through multiple pathways associated with the TME. First, FMT can potentially reconfigure the composition of immune cells in the TME. Accumulating evidence has conclusively demonstrated that following FMT, the TME consistently exhibits significantly enhanced infiltration of CD8+ T cells, Th1 cells, and antigen-presenting cells in responding patients. Concomitantly, a significant decrease is observed in the infiltration of myeloid-derived suppressor cells (MDSCs), RORγ+Th17 cells, and CD11b+CD11c+ suppressive myeloid cells within the TME following FMT intervention.^7,77–80^ Studies have indicated that following FMT, patients exhibit elevated levels of CD4+ and CD8+ T cells, while Tregs levels diminish.^81^ The ratio of intratumoral CD8+ T cell to Treg infiltration is elevated in patients following FMT.^82^ This modification in immune cell composition promotes the formation of an anti-tumor immune microenvironment. Specifically, CD8+ T cells function as the primary effector cells in anti-tumor immune responses, and their increased abundance can directly augment the ability to eliminate tumor cells.^83^ Th1 cells, conversely, indirectly potentiate anti-tumor immunity by releasing cytokines such as IFN-γ to stimulate macrophages and NK cells. The elevated number of antigen-presenting cells can facilitate the presentation of tumor antigens, thereby inducing more T cell activation. The diminished presence of MDSCs attenuates the suppression of effector T cells. The reduction in Tregs mitigates the suppression of effector T cells. These alterations following FMT collectively establish an immune microenvironment more favorable to the efficacy of ICIs.^78,79,84^

Second, FMT has the potential to augment the function of immune cells. Research has demonstrated that post-FMT, patients display enhanced activation states of mucosa-associated invariant T (MAIT) cells, characterized by elevated CD69 expression and reduced PD-1 expression.^85^ This alteration in activation state potentiates the immune surveillance function of MAIT cells. Furthermore, following FMT, the maturation and antigen presentation capacity of dendritic cells (DCs) are augmented.^15,86,87^ Mature DCs exhibit enhanced efficacy in presenting tumor antigens, thereby inducing more robust T cell activation.^79^ NK cell activity is potentiated following FMT.^88^ NK cells function as crucial anti-tumor effector cells in the innate immune system, and their augmented activity can directly intensify the cytotoxic effect on tumor cells.^43^ Additionally, FMT can facilitate the polarization of M1-type macrophages,^66,89^ which exhibit pro-inflammatory and anti-tumor properties.^90^

Furthermore, FMT has been demonstrated to regulate the expression of cytokines and chemokines. Research has demonstrated that serum levels of IFN-γ and IL-2 are elevated following FMT,^80^ and these cytokines exhibit significant antitumor effects. IFN-γ enhances the activity of CD8+ T cells, NK cells, and macrophages, while concurrently inhibiting tumor cell growth directly. Conversely, IL-2 stimulates the proliferation and activation of T cells and NK cells. Moreover, FMT has been shown to upregulate the expression of CXCL9 and CXCL10,^91,92^ chemokines that facilitate T cell recruitment into the TME.

Fourth, FMT has been shown to augment the efficacy of ICIs through the modulation of specific gut microbiota. Specifically, an increase in *Bifidobacterium* species has been demonstrated to enhance the efficacy of anti-PD-L1 therapy by promoting DCs functionality and facilitating the infiltration and accumulation of CD8+ T cells within the TME.^15,86^ Elevated levels of oral *Akkermansia muciniphila* have been shown to restore the anticancer effects of PD-1 blockade by facilitating the recruitment of CCR9+CXCR3+CD4+ T cells to the tumor site.^89,93,94^ Elevated abundance of *Faecalibacterium* exhibits a positive correlation with CD8+ T cell infiltration in tumors and a negative correlation with suppressive myeloid cells (CD11b+CD11c+). Furthermore, it may potentiate antitumor immune responses by augmenting antigen presentation and enhancing effector T cell function.^7,87^ *Bacteroides fragilis* facilitates the maturation of DCs and potentiates IL-12-dependent Th1 antitumor immune responses. Additionally, *Bacteroides fragilis* induces macrophage phenotype polarization toward M1 and upregulates the expression of CD80 and CD86 on these cells, thereby enhancing innate immunity.^66^ *Lactobacillus plantarum* significantly upregulates the expression of natural cytotoxicity receptor (NCR) proteins and enhances NK cell activation, consequently stimulating innate immunity.^66^ Studies in mouse models have demonstrated that *Lactobacillus rhamnosus* enhances immune responses by activating macrophages and NK cells, thereby initiating innate immunity.^88^

While FMT can enhance the efficacy of ICIs in numerous instances, it may not universally improve ICI efficacy across all clinical scenarios. Firstly, FMT may induce an expansion of specific immunosuppressive cell populations. Recent studies have demonstrated that following FMT, some patients exhibit increased proportions of Tregs and MDSCs within the TME.^20,55,95^ Tregs are known to suppress effector T cell function, whereas MDSCs exhibit broad immunosuppressive capabilities, inhibiting various immune cells, including T cells, NK cells, and DCs. The expansion of these immunosuppressive cell populations may potentially attenuate the antitumor efficacy of ICIs. Secondly, an expansion of specific gut microbial populations may suppress antitumor immune responses. For instance, research has demonstrated that an expansion of *Bacteroides* species is associated with increased Treg and MDSC populations, potentially resulting in diminished antigen presentation capacity and reduced lymphocyte infiltration within tumors.^20,87^ These alterations in the microbial community composition may compromise the therapeutic efficacy of ICIs. Thirdly, FMT may modulate the migratory patterns of specific immune cell populations. Research has revealed that post-FMT, some patients exhibit upregulation of the CCL2/CCR2 axis, resulting in enhanced myeloid cell migration to the liver.^96^ This alteration in cellular trafficking patterns may influence the therapeutic efficacy of ICIs in specific organ-localized tumors.^97^

In conclusion, the impact of FMT on ICI efficacy is multifaceted, encompassing both beneficial and potentially detrimental effects. The beneficial effects primarily manifest as enhanced effector T cell function, improved antigen presentation, modulation of favorable cytokine and chemokine expression profiles, and enrichment of beneficial microbial communities. The potentially detrimental effects are predominantly characterized by the expansion of immunosuppressive cell populations and other immunomodulatory alterations.

#### 3.1.5. Modulation of signaling pathways

Accumulating evidence indicates that the variations in ICI efficacy and prognosis following FMT are likely attributed to the modulation of various signaling pathways by the post-FMT gut microbiome. The beneficial effects of FMT on ICI efficacy are predominantly mediated through the modulation of TLR signaling pathways, resulting in amelioration of inflammatory conditions. A preclinical study has demonstrated that FMT can safely mitigate inflammation, with its mechanism of action primarily involving the TLR-MyD88-NF-κB signaling pathway.^98^ Among the various inflammatory and immune pathways modulated post-FMT, the restoration of TLR signaling pathway homeostasis (balance between pro-inflammatory pathways such as TLR4 signaling and anti-inflammatory pathways associated with TLR2 activation) represents a key advantage of FMT application in cancer.^99^ This homeostasis potentially facilitates the creation of a more favorable immune microenvironment, enhancing the efficacy of ICIs.

Conversely, the potential adverse effects of FMT on ICI efficacy warrant careful consideration. Initially, fecal samples from colorectal cancer patients utilized for FMT can potentially counteract the therapeutic effects of ICIs through the activation of signaling pathways that promote tumor growth or metastasis. In the mouse model, they demonstrated that the FMT with feces of colorectal cancer patients can induce the activation of the Wnt signaling pathway, which is instrumental in various stages of tumor development.^100^ Furthermore, investigations have revealed a mechanistic link between the post-FMT gut microbiome and cancer metastasis mediated by the IL-11/circRNA/miRNA axis.^101^ Additionally, certain FMTs can potentially compromise ICI efficacy through the activation of pro-inflammatory signaling pathways. Research has demonstrated that FMT from liver metastasis (LM) patients significantly enhances liver metastasis compared to FMT from HD.^96^ Following FMT with LM feces, plasma lipopolysaccharide (LPS) concentrations are elevated, resulting in the activation of the LPS/TLR4 signaling pathway, which plays a pivotal role in gut microbiome-mediated liver metastasis.^96^ This pro-inflammatory state can potentially disrupt the normal function of ICIs, consequently diminishing their therapeutic efficacy.

In conclusion, the impact of FMT on ICI efficacy is multifaceted and involves complex modulation of various signaling pathways. This intricate interplay potentially elucidates the heterogeneous responses to FMT in ICI therapy, ranging from enhanced efficacy to negligible changes or even adverse reactions. Future investigations should elucidate the regulatory mechanisms of various signaling pathways modulated by the post-FMT gut microbiome, potentially facilitating the development of more precise combination treatment strategies to optimize the synergistic anti-tumor effects of FMT and ICIs. Moreover, considering the complexity of the gut microbiome and inter-individual variability, the development of more sophisticated gut microbiome analysis and prediction models is imperative for improved prognostication and optimization of FMT’s impact on ICI efficacy in clinical settings.

### 3.2. Additional factors influencing FMT efficacy in ICI therapy

The efficacy of FMT in ICI therapy exhibits considerable heterogeneity, which can be attributed to the interplay of multiple complex factors.

Primarily, the success of FMT is intimately associated with transplantation frequency. Certain bacterial species may detach or exhibit resistance to cloning due to weak adhesion, resulting in bacterial cloning failure.^43^ The delivery method and frequency of FMT may be suboptimal, potentially impacting the efficacy of ICIs.^102^ Furthermore, donor selection is critical to the effectiveness of FMT. Research has demonstrated that FMT from different donors can result in markedly different clinical outcomes.^26^ Only responders possessing unique gut microbiome characteristics that distinguish them from non-responders are deemed suitable and efficacious candidates for FMT donors.^103^ The absence of microbial taxa necessary for anti-PD1 treatment response in FMT donors may lead to FMT failing to elicit a response to anti-PD1 therapy.^102^ Donor-related factors, including dietary habits and microbial diversity, may additionally impact the effectiveness of FMT.^60^ Specific dietary modifications (e.g., a diet high in dietary fiber) may facilitate the maintenance of beneficial microbiota, thus augmenting the synergistic effects of FMT and ICIs.^104^

Moreover, inter-individual variability among recipient patients may influence the efficacy of FMT. Strain tracking analysis has revealed inter-individual variations in the colonization of dominant donor microbes in recipients post-FMT, which appears unrelated to the response to anti-PD-1 immunotherapy.^105^ Certain recipients may fail to mount a response to tumors, irrespective of the microbiome composition, due to immunodeficiency or insufficient tumor immunogenicity.^54^ Of note, the efficacy of FMT may be associated with the recipient’s immune response to donor microbes. In patients exhibiting favorable responses, the post-FMT fecal microbiota demonstrates increased similarity to that of the donor, accompanied by a more pronounced induction of immunoglobulin G (IgG) antibodies against donor taxa.^102^ This immune response may contribute to augmenting the therapeutic efficacy of ICIs.

However, FMT may also present potential adverse consequences. For example, FMT from certain donors may introduce potentially carcinogenic bacteria or *polyketide synthase island (pks)+ Escherichia coli*, which may detrimentally impact patients.^106^ Furthermore, FMT may influence stromal cells and extracellular matrix barrier function, necessitating a balanced approach to mitigate potential negative impacts on treatment efficacy.^106^

In conclusion, the heterogeneity in FMT efficacy in ICI therapy can be attributed to the complex interplay of multiple factors, encompassing technical aspects of FMT, donor selection, inter-individual variability among recipients, microbiome-immune system interactions, potential risks, and patients’ dietary and lifestyle factors. Future research should aim to elucidate these factors to enhance the application of FMT in ICI therapy and improve the reproducibility and predictability of treatment outcomes.

## 4. Optimizing FMT strategies for enhanced ICI response

The successful implementation of FMT to enhance ICI efficacy requires comprehensive, systematic approaches for the selection and monitoring of optimal microbial communities. Recent clinical trials have demonstrated that specific microbial signatures substantially influence ICI outcomes. For instance, the clinical trial NCT03353402 revealed that treatment responders exhibited specific bacterial signatures, characterized by elevated abundances of *Enterococcaceae*, *Enterococcus*, and *Streptococcus australis*. A clinical trial (ChiCTR2100046768) investigating microsatellite stable metastatic colorectal cancer demonstrated that treatment responders harbored higher abundances of *Proteobacteria* and *Lachnospiraceae*, while exhibiting lower abundances of *Actinobacteria* and *Bifidobacterium*. Although the NCT03772899 trial reported a 65% ORR, the NCT03817125 trial demonstrated that Firmicutes-based preparation resulted in lower response rates compared to placebo, underscoring the complexity of composition-based selection. Understanding and optimizing these microbial signatures is crucial for developing robust FMT protocols that can consistently enhance ICI response rates. This section outlines critical approaches to microbiota selection and monitoring, focusing specifically on factors that directly influence immunotherapeutic efficacy.

### 4.1. Donor screening: the cornerstone of safety and efficacy

#### 4.1.1. Establishment of screening criteria

FMT has demonstrated significant promise in enhancing ICI therapy; however, the processes for donor screening and optimization continue to pose substantial challenges. Donor screening represents a crucial step in ensuring the safety and efficacy of FMT. Recent cases of pathogen transmission resulting from inadequate screening have underscored the critical importance of rigorous donor selection.^107,108^ A comprehensive array of factors must be meticulously evaluated when establishing screening criteria. Currently, individuals exhibiting high-risk behaviors, those with severe obesity, autoimmune diseases, malignancies, and neurological disorders are typically excluded from donor consideration.^109–111^ However, considerable debate persists within the academic community regarding the inclusion of factors such as age restrictions, BMI thresholds, family history of relevant diseases, or recreational drug use in the donor exclusion criteria. Furthermore, disparities in regional regulations and persistent uncertainties regarding the long-term safety of FMT exacerbate the challenges in establishing uniform standards.

#### 4.1.2. Optimization of screening methods

Optimal FMT screening protocols for patients receiving ICI therapy encompass multiple key components that surpass standard FMT protocols. The screening process adheres to a standardized three-tier approach: comprehensive medical history assessment, physical examination, and thorough laboratory testing. Laboratory screening encompasses both standard and advanced diagnostic panels. Standard screening includes analysis for common pathogens (*Clostridioides difficile*, norovirus, rotavirus), blood-borne viruses (HIV, hepatitis B and C), and antimicrobial-resistant organisms.

It is noteworthy that screening criteria for specific microorganisms remain a subject of ongoing debate in the scientific community. For example, while *Giardia* is generally recognized as potentially pathogenic, recent studies have demonstrated that FMT containing *Giardia* did not elicit significant adverse reactions in patients with rCDI.^112^ The viability of *Helicobacter pylori* in fecal samples remains a point of contention, resulting in diverse screening strategies adopted by various institutions.^113^ Moreover, the screening protocols for emerging pathogens necessitate continuous adaptation to address evolving threats. For instance, despite the absence of conclusive evidence supporting SARS-CoV-2 transmission via FMT, regulatory agencies have mandated its inclusion in screening protocols.^108^ The recent emergence of the monkeypox virus further underscores the necessity to enhance screening protocols for sexually transmitted infections.^108^

### 4.2. Selection of ICI response-enhancing microbiota: functional approaches

Optimizing FMT for ICI therapy necessitates a comprehensive understanding of the intricate interplay between the microbiome and tumor pathogenesis. Emerging evidence suggests that specific microorganisms can modulate tumor initiation and progression through diverse mechanisms, including direct interaction with host cells, induction of local inflammation, perturbation of immune homeostasis, and production of specific metabolites.^114^ Consequently, the selection of target microbiota should prioritize organisms with the potential to augment ICI efficacy. For instance, research has demonstrated that specific gut bacteria can potentiate ICI effects through mechanisms such as short-chain fatty acid production, T cell function modulation, or the TME alteration. This function-based selection strategy shows promise for enhancing the efficacy of FMT in ICI therapy.

### 4.3. Composition-based selection of ICI-Responsive microbial communities

Complementing functional screening, composition-based selection of microbiota represents another crucial strategy. This approach seeks to elucidate specific bacterial populations or consortia associated with favorable responses to ICI therapy. For example, investigations have revealed that the abundance of *Bacteroides* and *Bifidobacterium* genera exhibits a positive correlation with ICI efficacy. However, it is imperative to acknowledge the limitations of animal models in elucidating microbe-host interactions. Laboratory mice exhibit substantial differences in microbial composition compared to wild mice, and the immature immune system of germ-free mice may introduce bias into experimental results.^115^ This underscores the necessity for caution when extrapolating results from animal experiments to human subjects.

### 4.4. Monitoring and assessment of FMT-Enhanced ICI therapy

Within the framework of ICI therapy, the evaluation of FMT efficacy necessitates a multidimensional approach, encompassing: 1) Microbiome diversity indices, including but not limited to the Shannon index and Simpson index; 2) Quantitative alterations in the abundance of key bacterial populations; 3) Microbial function-related indicators, exemplified by short-chain fatty acid levels; 4) Immune system response parameters, including but not limited to T cell subpopulation proportions and functional characteristics; 5) Clinical outcome metrics, encompassing tumor response rate, PFS, and OS. Moreover, meticulous surveillance of potential adverse reactions, particularly infections and autoimmune responses, is imperative. The establishment of standardized reporting systems, exemplified by the American Gastroenterological Association (AGA) and European FMT registries, will facilitate a more comprehensive understanding of the short- and long-term effects of FMT.^116^ It is crucial to acknowledge that, beyond infectious complications, FMT may potentially pose theoretical long-term risks, including but not limited to the induction of autoimmune diseases, neurological disorders, or metabolic disturbances.^117^ While current evidence does not conclusively substantiate these risks, continued vigilance and rigorous monitoring are essential during long-term follow-up.

The identification and optimization of gut microbiota for ICI therapy presents a complex and challenging undertaking. While FMT has demonstrated promise in augmenting ICI efficacy, it continues to encounter numerous challenges, encompassing the refinement of donor screening criteria, enhancement of preparation methodologies, personalization of administration protocols, and evaluation of long-term safety profiles. Future investigations should prioritize the following aspects: 1) Enhancing donor screening criteria and detection methodologies, with particular emphasis on emerging pathogens; 2) Innovating novel FMT formulations to improve stability and optimize controllability; 3) Conducting comprehensive investigations into the interaction mechanisms between the microbiome and host immune system to establish a robust theoretical foundation for targeted interventions; 4) Elucidating personalized FMT strategies, taking into account patients’ microbiome profiles, tumor classifications, and immune status; 5) Implementing long-term follow-up protocols to assess the prolonged safety and efficacy of FMT in ICI therapy.

In general, FMT has exhibited favorable safety profiles across diverse populations, including pediatric, immunosuppressed, and cirrhotic patients.^118–121^ Nevertheless, additional empirical evidence is required for severely immunocompromised individuals. Adherence to rigorous donor screening and monitoring protocols can effectively mitigate most potential risks. Through intensified research efforts and technological advancements, the development of safer and more efficacious FMT protocols may yield novel breakthroughs in cancer immunotherapy. Realizing this objective necessitates close collaboration among clinicians, microbiologists, and immunologists, coupled with active engagement from regulatory agencies, to collectively advance the standardized application of FMT in ICI therapy.

## 5. Future perspectives

**Figure 3.**
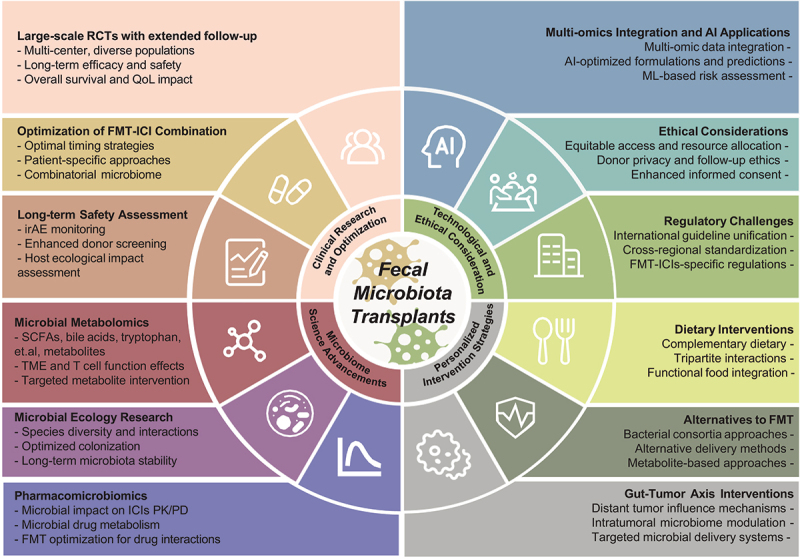
Future research directions for the combination therapy of fecal microbiota transplantation (FMT) and immune checkpoint inhibitors (ICIs).

The integration of FMT into ICI therapy has exhibited significant promise while simultaneously encountering multiple challenges. This section delineates future directions for the combinatorial approach of FMT and ICIs from multiple perspectives, offering insights and research prospects in this nascent field (Figure 3).

This figure delineates 12 critical future research directions for the combination therapy of FMT and ICIs. These directions span a broad spectrum of areas from basic research to clinical applications, underscoring the complexity and potential of this field. Primarily, the implementation of large-scale, multi-center randomized controlled trials will yield more robust clinical evidence for FMT-ICIs combination therapy. Subsequently, optimizing the combination strategy of FMT and ICIs, encompassing the timing of administration, frequency, and development of personalized regimens, will facilitate the maximization of therapeutic effects. Comprehensive long-term safety and potential risk assessment are essential for safeguarding the efficacy and safety of the treatment. Investigations into the interaction between microbial metabolites and ICIs treatment will elucidate the underlying therapeutic mechanisms. Microbial community ecology research will unveil the correlation between community structure and function and treatment efficacy. Pharmacomicrobiomics research will delineate the impact of gut microbiota on the pharmacokinetics and pharmacodynamics of ICIs. Intervention studies targeting gut microbiota and tumor microenvironment will investigate the possibility of distal regulation of the tumor microenvironment. Enhancing FMT efficacy through dietary interventions represents a promising research avenue. The integration of multi-omics approaches and application of artificial intelligence will offer powerful tools for precision medicine. Addressing regulatory challenges will facilitate the standardization and implementation of FMT-ICIs combination therapy. Ethical considerations will safeguard the fairness and accessibility of this innovative treatment modality. The holistic advancement of these research directions will significantly propel the development of FMT-ICIs combination therapy, offering renewed hope to cancer patients. Ultimately, the exploration of alternatives to FMT, including defined bacterial consortia and metabolite-based approaches, represents an emerging frontier in microbiome-based immunotherapy.

FMT: Fecal Microbiota Transplantation; ICIs: Immune Checkpoint Inhibitors.

### 5.1. The Necessity of Large-scale, multi-center randomized controlled trials

Well-designed, large-scale, multi-center randomized controlled trials (RCTs) are essential to comprehensively evaluate the therapeutic potential of FMT in augmenting ICI therapy. These trials should encompass diverse tumor types, stages, and patient populations across ethnicities and geographical regions, while maintaining stringent inclusion and exclusion criteria. Critical parameters requiring systematic investigation include optimal administration protocols, dosage regimens, frequencies, and timing of FMT-ICI combination, as well as standardized preparation methods for fecal material. Furthermore, long-term follow-up studies are crucial for assessing sustained efficacy and safety profiles, focusing on both primary and secondary endpoints such as OS, PFS, ORR, and quality of life.^108^

### 5.2. Optimization of combined use of FMT and ICIs

The optimization of FMT-ICI combination therapy necessitates rigorous investigation into the optimal timing of administration and the development of personalized treatment regimens based on scientific evidence. Given the heterogeneity of patient microbiomes, protocols should require customization based on baseline microbiome profiles, tumor characteristics, host genetic factors, and immunological status. This approach encompasses standardized donor selection, personalized FMT formulations, and integration with complementary microbiome modulation strategies, including prebiotics and dietary interventions

Although autologous FMT has demonstrated therapeutic potential in some clinical settings, especially in microbiome restoration following antibiotic treatment,^122^ to date, there are no published clinical studies specifically evaluating the combination of autologous FMT with ICI therapy. This knowledge gap presents a critical avenue for future research, specifically in patients who exhibit initial therapeutic responses to ICIs but subsequently develop resistance.

### 5.3. Long-term safety and potential risk assessment

With the escalating utilization of FMT in ICI therapy, the evaluation of its long-term safety and potential risks assumes paramount importance. It is imperative to design long-term follow-up studies that facilitate the systematic collection and analysis of patients’ clinical data, microbiome alterations, and potential adverse events. Special emphasis should be placed on elucidating the impact of FMT on immune-related adverse events (irAEs), encompassing potential exacerbation or mitigation effects. In the context of irAE alleviation or improvement, emerging evidence suggests that FMT may have therapeutic potential for ICI-induced colitis.^123^ Concurrently, it is essential to develop more comprehensive strategies for potential risk identification and prevention. These strategies should encompass the refinement of donor screening methods, optimization of FMT preparation and storage processes, and the development of safer administration routes.

### 5.4. Microbial metabolites and ICI therapy

Microbial metabolites have emerged as critical mediators orchestrating immune responses and modulating ICI efficacy. Future investigations should elucidate the mechanisms by which key metabolites, specifically SCFAs, bile acids, and tryptophan metabolites, regulate the TME, T cell functionality, and ICI-mediated responses. This mechanistic understanding may enable the transition from conventional FMT to precision-guided metabolic interventions, potentially advancing therapeutic efficacy. Comprehensive metabolomic profiling of patient-derived specimens (feces, blood, and tumor tissues) will be essential for elucidating FMT-mediated effects on ICI efficacy through metabolic regulation, potentially identifying novel predictive biomarkers of therapeutic response.^124^

### 5.5. Microbial community ecology research

The strategic optimization of microbial communities constitutes a fundamental research priority. Although enhanced microbial diversity achieved through multi-donor approaches may augment ICI efficacy, conclusive evidence comparing the therapeutic outcomes of single versus multi-donor FMT remains insufficient.^125,126^ Future investigations should systematically evaluate whether specific bacterial species or defined microbial consortia more effectively modulate ICI outcomes, while assessing diverse community integration strategies.^127^

Elucidating the interactions between transplanted and resident microbiota is essential, requiring refined gut preparation protocols and optimized antibiotic pretreatment regimens.^128^ The development of robust methodologies for evaluating donor bacterial engraftment and quantifying recipient microbiota composition remains fundamental.^127^ Furthermore, investigating microbial community stability and resilience following FMT is critical for optimizing therapeutic protocols. Advanced bioinformatics and mathematical modeling approaches may facilitate the prediction of community dynamics and guide the development of personalized therapeutic strategies.^108^

### 5.6. Pharmacomicrobiomics research

As our understanding of the interactions between gut microbiota and drugs continues to deepen, pharmacomicrobiomics is emerging as a crucial branch of FMT and ICI research. There is a pressing need for in-depth investigations into how gut microbiota affect the pharmacokinetics and pharmacodynamics of ICIs and other antitumor drugs. This encompasses the influence of microorganisms on drug metabolism, absorption, and distribution, as well as the modulation of drug action by microbial metabolites.^129–131^ For instance, alterations in the microbiome composition may affect the pharmacokinetics of drugs such as levodopa, attributed to the influence of bacterial tyrosine decarboxylase on levodopa metabolism.^130,131^ Moreover, anticancer drugs have been demonstrated to interact with microorganisms.^132^ Future research should elucidate how FMT affects the pharmacokinetics, pharmacodynamics, and metabolism of drugs in various organs, particularly the kidneys and liver. Additionally, further research is needed to elucidate how FMT can facilitate the rational modification of gut microbiota, thereby fostering beneficial host-microbiome-drug interactions for the recipient.

### 5.7. Intervention from gut microbiota to tumor microenvironment

Future research should prioritize the investigation of the gut-tumor axis, particularly the mechanisms by which gut microbiota influence the microenvironment of distant tumors through various pathways. In-depth investigations are required to elucidate how metabolites, cytokines, and other signaling molecules produced by gut microbiota traverse blood circulation to reach tumor sites and subsequently modulate the functions of tumor cells, immune cells, and stromal cells. This systematic research approach will facilitate a comprehensive understanding of the mechanisms by which FMT influences the efficacy of ICIs. Furthermore, targeted intervention of intratumoral microbiota represents an emerging research direction. Accumulating evidence suggests that tumor tissues harbor distinct microbial communities, which may directly influence tumor growth, metastasis, and response to treatment. Future studies should explore methods to modulate intratumoral microbiota through FMT or other approaches to enhance the antitumor effects of ICIs. This endeavor may necessitate the development of novel administration routes, such as local injection or targeted delivery systems.

### 5.8. Further enhancing FMT efficacy through dietary interventions

Given its pivotal role in shaping the gut microbiome, the impact of diet on the combined therapy of FMT and ICIs merits comprehensive investigation. Future research endeavors should elucidate how dietary interventions can optimize FMT outcomes and potentiate ICI efficacy. This approach may necessitate the design of specific dietary regimens (e.g., high-fiber diets, Mediterranean diets, or anti-inflammatory diets) to foster the proliferation of beneficial bacteria and augment the production of metabolites. Concurrently, it is imperative to investigate the interactions among diet, FMT, and ICIs to formulate optimal comprehensive treatment strategies.^133–135^ For instance, extant research indicates that anti-inflammatory diets combined with FMT correlate with favorable responses in ulcerative colitis (UC).^133,134^ FMT in conjunction with fermentable fibers demonstrated enhanced insulin sensitivity in patients with metabolic syndrome (MetS).^135^ Furthermore, the integration of functional foods and nutritional supplements into the combined therapy of FMT and ICIs represents a promising avenue for future research. Potential candidates include specific prebiotics, polyphenolic compounds, and precursors of SCFAs.

### 5.9. Integration of Multi-omics and application of artificial intelligence

The rapid advancement of high-throughput sequencing technologies and bioinformatics has positioned integrated multi-omics analysis to assume an increasingly pivotal role in FMT and ICI research. Future research efforts should focus on integrating diverse omics data, encompassing metagenomics, transcriptomics, metabolomics, and proteomics, to elucidate comprehensively the impact of FMT on host-microbe-tumor interactions. This systems biology approach has the potential to uncover novel biomarkers and therapeutic targets, thereby establishing a foundation for personalized treatment. The implementation of artificial intelligence (AI) and machine learning technologies in this field is anticipated to intensify. These advanced computational methods can facilitate the extraction of key information from massive multi-omics data, enable the prediction of treatment responses, and contribute to the optimization of treatment strategies. For instance, AI can be employed to design optimal FMT formulations, forecast patient responses to ICIs, and identify potential adverse reaction risks.^108,136^ However, it is crucial to acknowledge the limitations and potential biases inherent in AI applications to ensure their judicious implementation in clinical decision-making.

### 5.10. Regulatory challenges

The clinical implementation of FMT in combination with ICI therapy presents significant regulatory and ethical challenges that require careful consideration. Regulatory frameworks for FMT demonstrate substantial variation across jurisdictions, with some countries classifying it as a biological product, while others categorize it as tissue transplantation or a pharmaceutical agent.^108^ These classification disparities significantly influence production standards, storage protocols, and clinical applications, thereby affecting the implementation of FMT in ICI therapy.^137,138^ The establishment of a harmonized international regulatory framework is essential to standardize FMT applications in ICI therapy while maintaining sufficient flexibility to accommodate scientific advances and ensuring patient safety.

### 5.11. Ethical considerations

The ethical landscape of FMT-ICI combination therapy presents unique challenges, specifically in relation to informed consent and donor considerations. The complexity of this combined treatment modality, encompassing its unknown long-term effects and potential risks,^108^ necessitates a rigorous and comprehensive informed consent process. Donor screening protocols must be adapted to address ICI therapy-specific requirements, including evaluation of immune status and tumor history. The protection of donor privacy, especially concerning long-term health status changes, requires systematic evaluation and implementation. A multidisciplinary, international ethics committee comprising medical experts, ethicists, legal professionals, patient advocates, and policymakers could help address these challenges. Additionally, enhanced public education efforts are crucial for improving societal understanding and acceptance of FMT-ICI combination therapy.^139,140^

### 5.12. The investigation into alternatives to FMT

Research into alternatives to FMT represents a critical frontier in advancing microbiome-based immunotherapy. Although FMT demonstrates therapeutic potential, precisely defined bacterial consortia provide more standardized and controllable therapeutic approaches. Emerging evidence indicates that strategically designed bacterial combinations demonstrate efficacy in modulating immune responses and enhancing ICI therapeutic outcomes. For instance, a phase 2 clinical trial investigating Microbial Ecosystem Therapeutic 4 (MET4), a defined consortium of bacterial strains, in combination with ICIs showed encouraging preliminary safety results and immune modulation in patients with advanced solid tumors.^141^ Alternative delivery methods are also gaining attention, with studies showing that freeze-dried, encapsulated bacterial preparations can maintain therapeutic efficacy while improving stability and ease of administration.^142^ Metabolite-based approaches have emerged as another promising alternative, as demonstrated by recent research showing that indole-3-carboxaldehyde (3-IAld), a microbial tryptophan metabolite, can optimize ICI therapy by protecting against ICI-induced colitis while maintaining antitumor efficacy through modulation of both host immunity and microbiota composition.^143^ These alternative approaches could provide more precise and controlled methods for microbiome modulation in cancer immunotherapy while potentially reducing the safety concerns associated with traditional FMT.

## 6. Conclusion

The integration of FMT into ICI therapy presents broad prospects while simultaneously facing numerous challenges and opportunities. To address the controversial effects of FMT in ICIs and elucidate its potential mechanisms, future research should delve into the interaction mechanisms between FMT and ICIs at multiple levels, optimize combination treatment strategies, ensure long-term safety, and aim to realize the clinical benefits of FMT in enhancing ICI efficacy. Large-scale clinical trials, microbial community ecology studies, metabolomics analyses, multi-omics integration, and AI applications are poised to be key drivers in advancing this field. Concurrently, it is imperative to foster interdisciplinary collaboration, seamlessly integrating knowledge and technologies from microbiology, immunology, oncology, and bioinformatics to comprehensively augment the application of FMT in ICI therapy. Despite the numerous challenges confronting FMT, it is projected to remain a crucial tool for elucidating the role of the microbiome in health and disease. The hypotheses and associative findings emanating from FMT research are expected to continue propelling mechanistic studies and the development of novel microbial therapies. As research advances, the aim is to develop more precise and effective FMT-ICI combination treatment strategies, offering renewed hope to cancer patients. In this rapidly evolving field, it is essential to maintain an open and innovative mind-set while advancing research cautiously and responsibly to guarantee that the ultimate clinical applications are both safe and efficacious.
